# [Ni^III^(OMe)]-mediated reductive activation of CO_2_ affording a Ni(κ^1^-OCO) complex†
†Electronic supplementary information (ESI) available. CCDC 785531, 1435237 and 1435238. For ESI and crystallographic data in CIF or other electronic format see DOI: 10.1039/c5sc04652a


**DOI:** 10.1039/c5sc04652a

**Published:** 2016-02-24

**Authors:** Tzung-Wen Chiou, Yen-Ming Tseng, Tsai-Te Lu, Tsu-Chien Weng, Dimosthenes Sokaras, Wei-Chieh Ho, Ting-Shen Kuo, Ling-Yun Jang, Jyh-Fu Lee, Wen-Feng Liaw

**Affiliations:** a Department of Chemistry , National Tsing Hua University , Hsinchu , 30013 , Taiwan . Email: d9623817@oz.nthu.edu.tw ; Email: wfliaw@mx.nthu.edu.tw; b Department of Chemistry , Chung Yuan Christian University , Taoyuan , 32023 , Taiwan; c SLAC National Accelerator Laboratory , Menlo Park , CA 94025 , USA; d Department of Chemistry , National Taiwan Normal University , Taipei , 10610 , Taiwan; e National Synchrotron Radiation Research Center , Hsinchu , 30013 , Taiwan

## Abstract

Carbon dioxide is expected to be employed as an inexpensive and potential feedstock of C_1_ sources for the mass production of valuable chemicals and fuel. Versatile chemical transformations of CO_2_, *i.e.* insertion of CO_2_ producing bicarbonate/acetate/formate, cleavage of CO_2_ yielding μ-CO/μ-oxo transition-metal complexes, and electrocatalytic reduction of CO_2_ affording CO/HCOOH/CH_3_OH/CH_4_/C_2_H_4_/oxalate were well documented. Herein, we report a novel pathway for the reductive activation of CO_2_ by the [Ni^III^(OMe)(P(C_6_H_3_-3-SiMe_3_-2-S)_3_)]^–^ complex, yielding the [Ni^III^(κ^1^-OCO˙^–^)(P(C_6_H_3_-3-SiMe_3_-2-S)_3_)]^–^ complex. The formation of this unusual Ni^III^(κ^1^-OCO˙^–^) complex was characterized by single-crystal X-ray diffraction, EPR, IR, SQUID, Ni/S K-edge X-ray absorption spectroscopy, and Ni valence-to-core X-ray emission spectroscopy. The inertness of the analogous complexes [Ni^III^(SPh)], [Ni^II^(CO)], and [Ni^II^(N_2_H_4_)] toward CO_2_, in contrast, demonstrates that the ionic [Ni^III^(OMe)] core attracts the binding of weak σ-donor CO_2_ and triggers the subsequent reduction of CO_2_ by the nucleophilic [OMe]^–^ in the immediate vicinity. This metal–ligand cooperative activation of CO_2_ may open a novel pathway promoting the subsequent incorporation of CO_2_ in the buildup of functionalized products.

## Abstract 2

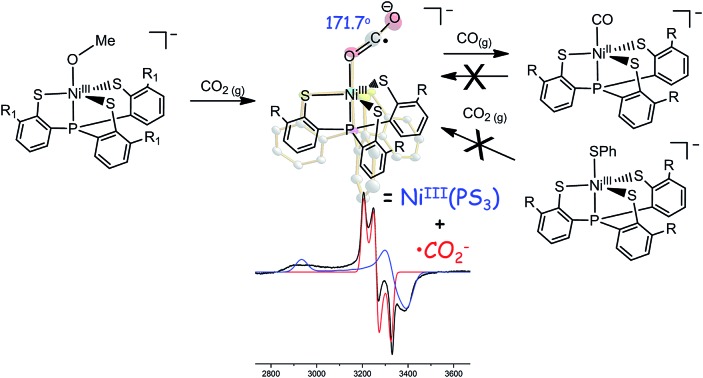
We report a novel pathway for the reductive activation of CO_2_ by the [Ni^III^(OMe)(P(C_6_H_3_-3-SiMe_3_-2-S)_3_)]^–^ complex, yielding the [Ni^III^(κ^1^-OCO˙^–^)(P(C_6_H_3_-3-SiMe_3_-2-S)_3_)]^–^ complex.

## Introduction

Carbon dioxide, the waste from human activity embodying the nature of high thermodynamic stability and chemical inertness, is expected to be employed as an inexpensive and potential feedstock of C_1_ sources for the regeneration of valuable chemicals and fuel.^1^,^2^ Nature developed carbon monoxide dehydrogenase (CODH) to harbor a Ni–Fe cluster for the reversible interconversion between CO_2_ and CO.^3^–^5^ To gain insight into the mechanism for the conversion of CO_2_ to CO in CODH, several Ni–CO_2_ adducts derived from the reaction of a low-valent Ni complex and CO_2_ were reported.^6^–^9^ The direct electrochemical reduction of CO_2_ affords oxalate, carbon monoxide, formic acid, methanol, methane, and ethylene.^10^ To gain insight into the transformation of CO_2_ at a molecular level, the chemistry of the activation of CO_2_*via* nucleophilic attack/interaction on the polarized C center, in addition to the reduction of the coordinated CO_2_ ligand by low-valence transition metal complexes, has grown explosively over past years.^11^ The versatile chemical transformations of CO_2_, *i.e.* insertion of CO_2_ producing bicarbonate/acetate/formate,^12^–^18^ cleavage of CO_2_ yielding μ-CO/μ-oxo transition-metal complexes,^19^–^23^ reduction of CO_2_ affording CO/HCOOH/CH_3_OH/CH_4_/C_2_H_4_/C_2_H_6_/methylene, and electrocatalysis of CO_2_ converting it into oxalate, were well documented.^24^–^26^ The direct electrochemical reduction or electrocatalytic transformation of CO_2_ for the mass production of valuable chemicals and fuel, however, relies on the consumption of sustainable electric potential energy. Here we show a novel pathway for the reductive activation of CO_2_ by a mononuclear Ni(iii) complex [Ni^III^(OMe)(P(C_6_H_3_-3-SiMe_3_-2-S)_3_)]^–^.^27^ This [Ni^III^-(OMe)]-mediated reduction of CO_2_ yields the complex Ni^III^(κ^1^-OCO˙^–^), evidenced by single-crystal X-ray diffraction, EPR, SQUID, Ni/S K-edge X-ray absorption spectroscopy, IR and Ni valence-to-core X-ray emission spectroscopy. The ionic [Ni^III^(OMe)] core provides a kinetic pathway to induce the binding of CO_2_ and trigger the subsequent reduction of CO_2_ by the nucleophilic [OMe]^–^ in the immediate vicinity. The covalent [Ni^III^(SPh)] core as well as Ni(ii) center in complexes [Ni^II^(L)(P(C_6_H_3_-3-SiMe_3_-2-S)_3_)]^–^ (L = CO or N_2_H_4_), in contrast, are inert toward CO_2_.^28^

## Results and discussion

### Synthesis and characterization of nickel κ^1^-OCO complex

**Scheme 1:**
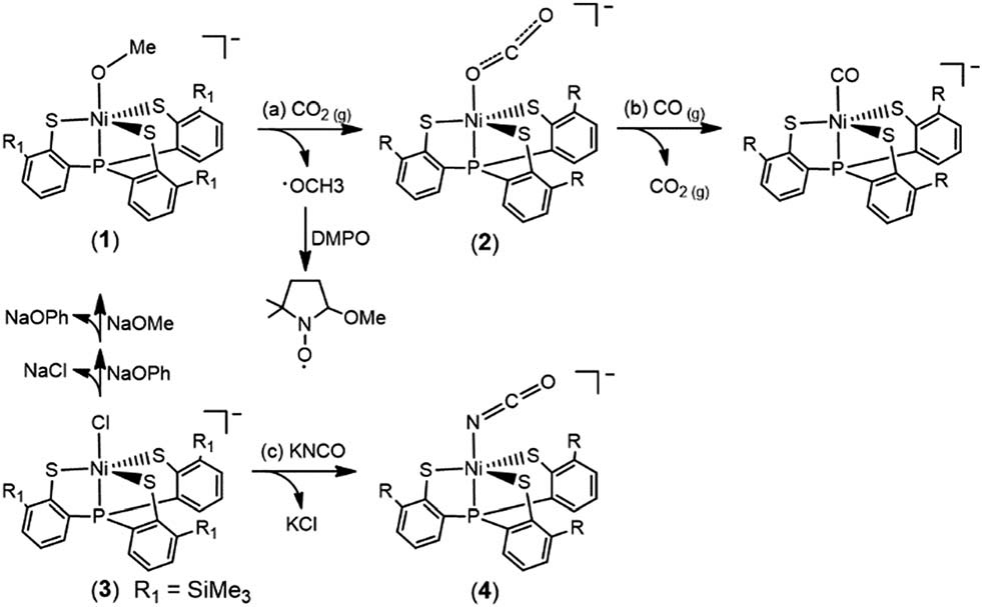


When CO_2(g)_ was bubbled into the thermally stable [Ni^III^(OMe)(PS_3_)]^–^ (**1**) (PS_3_ = P(C_6_H_3_-3-SiMe_3_-2-S)_3_) in THF,^27^ a pronounced color change from blue green to yellow green occurred to yield the O-bound κ^1^-CO_2_ complex [Ni(κ^1^-OCO)(PS_3_)]^–^ (**2**), instead of complexes [Ni(OC(O)OCH_3_)-(PS_3_)]^–^ or [Ni(OC(O)H)(PS_3_)]^–^*via* the classical insertion or β-H migration mechanisms (Scheme 1a).^9^,^12^–^16^

**Fig. 1:**
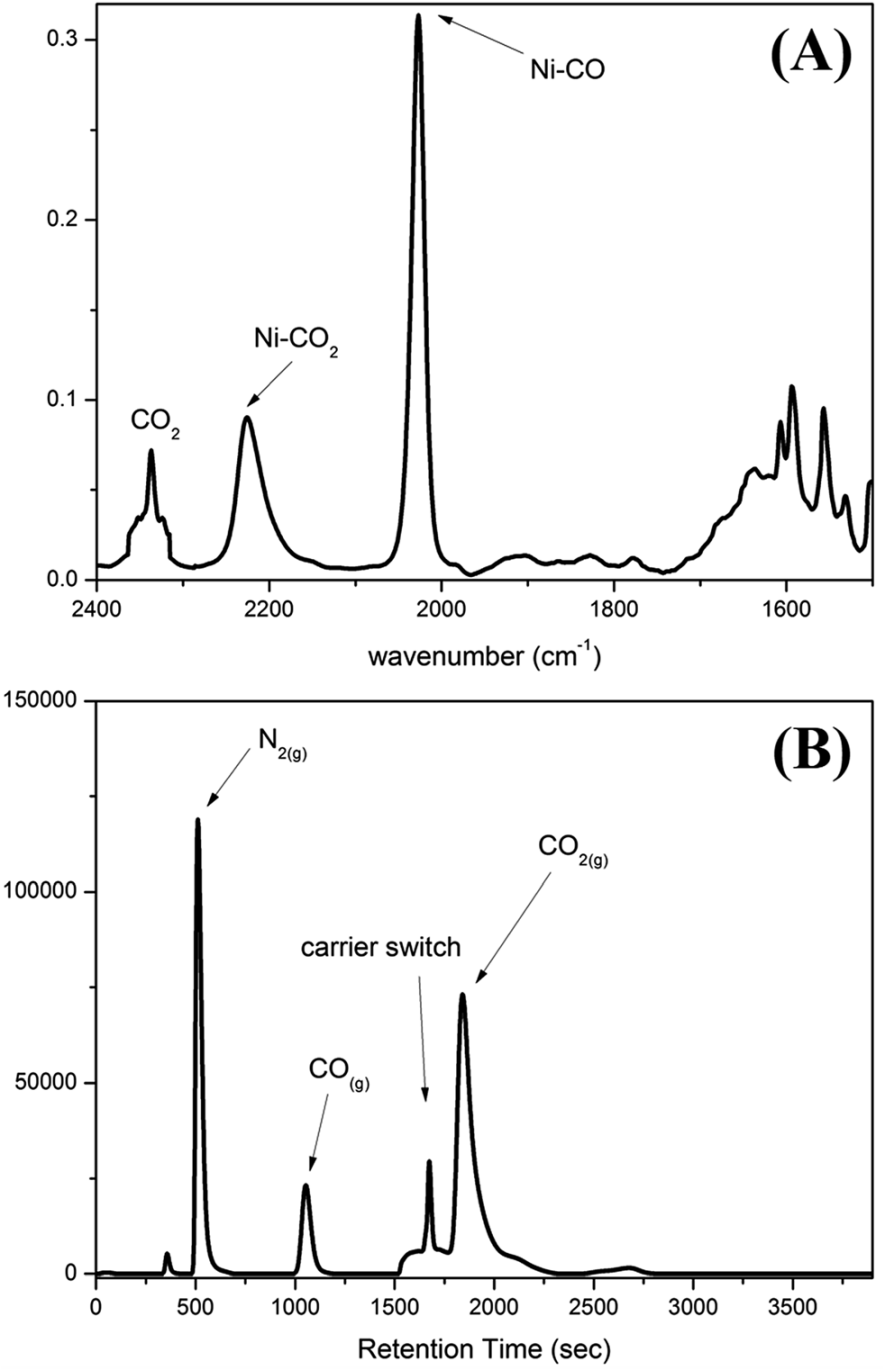
(A) IR spectra for the transformation of complex **2** to [Ni^II^(CO)(PS_3_)]^–^ in THF. The decrease in the intensity of the IR *ν*_CO_2__ peak at 2226 cm^–1^ exhibited by complex **2** with the simultaneous formation of an IR *ν*_CO_ peak at 2027 cm^–1^ indicated the formation of complex [Ni^II^(CO)(PS_3_)]^–^. (B) GC chromatogram, derived from the sample collected from the headspace of the tube containing the reaction solution of complex **2** and CO_(g)_, indicating the release of CO_2(g)_ during the transformation of complex **2** to [Ni^II^(CO)(PS_3_)]^–^.

The accompanied formation of [˙OMe] in the reaction described above was corroborated using the spin-trapping reagent DMPO (ESI Fig. S1†).^29^ The IR *ν*_OCO_ stretching peak at 2177 cm^–1^ (KBr) (*ν*_OCO_: 2226 cm^–1^ in THF) exhibited by complex **2** supports the formation of [Ni(κ^1^-OCO)(PS_3_)]^–^, which is consistent with the isotopic shift of the IR *ν*_OCO_ stretching peak to 2117 cm^–1^ (KBr) observed in the ^13^CO_2_ labeling experiment (ESI Fig. S2†). The conversion of complex **1** to complex **2** under a CO_2_ atmosphere was also monitored by UV-vis spectrometry; the intense bands at 419 and 605 nm disappeared with the simultaneous formation of absorption bands at 425 and 610 nm (THF) (ESI Fig. S3†). The green needle crystals of complex **2** were isolated when complex **2** was recrystallized from THF–diethyl ether at room temperature. As shown in Scheme 1b, treatment of complex **2** with CO_(g)_ led to the formation of the reported complex [Ni^II^(CO)(PS_3_)]^–^ accompanied by the release of CO_2(g)_ characterized by IR and GC (Fig. 1).^28^

**Fig. 2:**
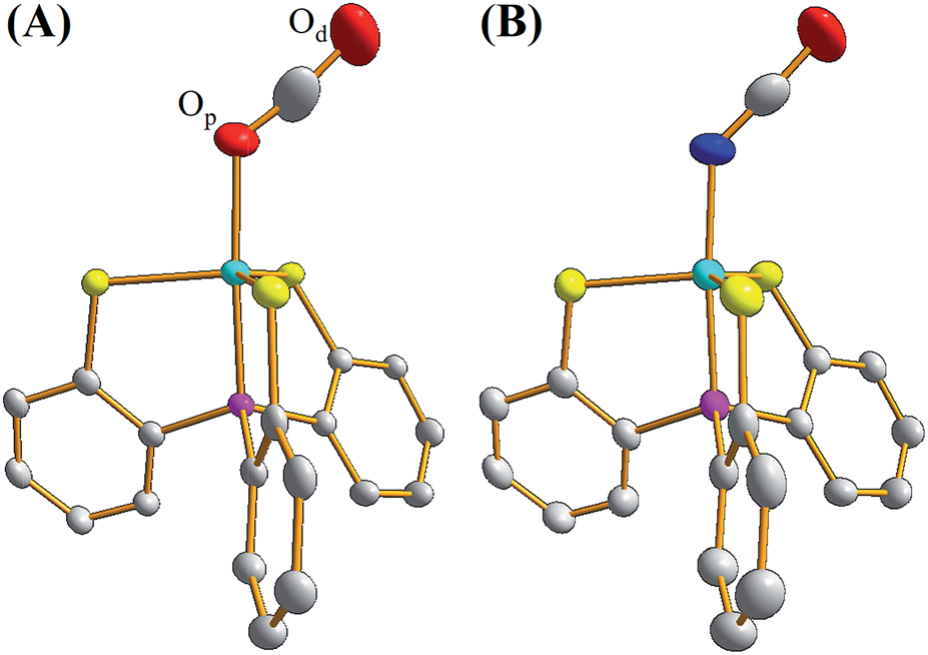
ORTEP drawing schemes of (A) complex **2** and (B) complex **4** with thermal ellipsoids drawn at a 50% probability level. The Ni, P, S, O, N, and C atoms are shown as light blue, purple, yellow, red, blue, and white ellipsoids. The H atom and TMS group are omitted for clarity. Selected bond distances (Å) and angles (°) for complex **2**: Ni–O_p_, 2.028(3); Ni–P, 2.122(1); Ni–S, 2.221(1), 2.287(1), and 2.285(1); O_p_–C, 1.132(6); O_d_–C, 1.240(7); O–Ni–P, 176.2(1); Ni–O–C, 127.0(4); O_p_–C–O_d_, 171.7(7). Selected bond distances (Å) and angles (°) for complex **4**: Ni–N, 1.933(2); Ni–P, 2.120(1); Ni–S, 2.226(1), 2.286(1), and 2.291(1); O–C, 1.200(3); N–C, 1.181(3); N–Ni–P, 175.0(1); Ni–N–C, 135.1(2); O–C–N, 177.2(3).

To contrast complex **2** containing a [Ni^III^:CO_2_˙^–^] or [Ni^II^:CO_2_] center, complex [Ni^III^(NCO)(PS_3_)]^–^ (**4**) was synthesized *via* the reaction of [Ni(Cl)(PS_3_)]^–^ (**3**) and [K][NCO] to mimic the isolobal [Ni^III^:CO_2_] (Scheme 1c). Fig. 2 displays ORTEP plots of complexes **2** and **4**, with the selected bond distances and angles given in the caption. The strain effect of the chelating ligand ([PS_3_]^3–^) in the coordination sphere of complexes **2** and **4** explains that the Ni is in a distorted trigonal bipyramidal geometry with three thiolates locating equatorial positions and the phosphorus is occupying an axial position trans to the [OCO] and [NCO] ligands. In contrast to the linear N–C–O (177.2(3)°) bond observed in complex **4**, complex **2** displays a bent O–C–O bond with a bond angle of 171.7(7)°. Compared to the similar O–C and N–C bond distances of 1.200(3) and 1.181(3) Å in complex **4**, the dramatic difference (∼0.1 Å) in O–C bond lengths, 1.132(6) *vs.* 1.240(7) Å, found in complex **2** moreover indicates the polarization of CO_2_*via* reductive activation affording a [Ni^III^:CO_2_˙^–^] species.^22^,^30^,^31^ A similar polarization of CO_2_ was reported in the O-bound κ^1^-CO_2_ coordinated complex [((^Ad^ArO)_3_tacn)-U^IV^(CO_2_˙^–^)] ((^Ad^ArOH)_3_tacn = 1,4,7-tris(3-adamantyl-5-*tert*-butyl-2-hydroxybenzyl)-1,4,7-triazacyclononane), O–C = 1.122(4) and 1.277(7) Å, with the linear U–O–C and O–C–O bonds stabilized by the sterically encumbering ligand framework.^30^,^31^ Besides, complex **2** displays a significantly longer Ni–O bond distance (2.028(3) Å) than those observed in [Ni^II^(L)(pyN_2_^Me2^)]^1–^ complexes (1.857(5) Å for L = HCO_2_^–^; 1.817(4) Å for L = HCO_3_^–^).^32^

### X-ray absorption/emission spectrum

**Fig. 3:**
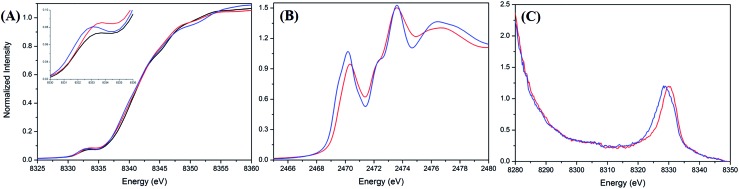
(A) Ni K-edge X-ray absorption spectra of complexes **1** (black), **2** (red), and **4** (blue). (B) S K-edge X-ray absorption and (C) Ni valence-to-core X-ray emission spectra of complexes **2** (red) and **4** (blue).

A Ni and S K-edge X-ray absorption spectroscopic (XAS) study of complex **2** was further attempted to investigate its electronic structure using complexes **1** and **4** as reference complexes. As shown in Fig. 3A, the Ni K-edge XAS of complex **2** (8333.1 eV) together with analogous complexes **1** (8332.9 eV) and **4** (8332.7 eV) shows a similar Ni_1s_-to-Ni_3d_ transition energy. Accordingly, the formal oxidation state of Ni in complex **2** is similar to those of complexes **1** and **4**, which are generally known as a L ligand bound to a d^7^ Ni(iii) center in [Ni^III^(L) (PS_3_)]^–^.^28^Fig. 3B and ESI Fig. S4† depict the S K-edge XAS spectra of complexes **1**, **2**, and **4**, whereas ESI Fig. S5† shows the calculated S K-edge XAS spectra and spectral deconvolution. As shown in Table 1, the intensity-weighted average energy of the S_1s_-to-Ni_3d_ transitions in combination with the S_1s_-to-S*C–S transition energy demonstrate that the Ni_3d_ manifold orbital energy of complex **2** is 0.3 eV higher than those of complexes **1** and **4**.^33^,^34^ With further regard to the Ni_1s_-to-Ni_3d_ pre-edge transition energy observed in the Ni K-edge XAS, Ni *Z*_eff_ of complexes **1**, **2**, and **4** are all comparable. That is, the Ni and S K-edge XAS study supports the [Ni^III^:CO_2_˙^–^] electronic structure in complex **2**. As shown in Fig. S6,† the cyclic voltammogram of a 2 mM solution of complex **4** in CH_3_CN indicates a reversible interconversion between Ni^III^/Ni^II^ at *E*_1/2_ = –0.58 V and an irreversible oxidation at *E*_pa_ = –0.21 V, whereas complex **2** exhibits a reversible interconversion between Ni^III^/Ni^II^ at *E*_1/2_ = –0.70 V and an irreversible oxidation at *E*_pa_ = –0.29 V (*vs.* Fc/Fc^+^).

**Table 1:** Ni_1s_ → Ni_3d_, S_1s_ → Ni_3d_, S_1s_ → S*C–S transition energy and S_1s_ → Ni_3d_ transition intensity of complexes **1**, **2**, **4**, and [Ni(SPh)(PS_3_)]^–^, derived from the Ni and S K-edge X-ray absorption spectroscopy

Complexes	Ni_1s_ → Ni_3d_ energy^*a*^ (eV)	S_1s_ → Ni_3d_ energy^*b*^ (eV)			S_1s_ → Ni_3d_ intensity^*b*^		S_1s_ → S*C–S energy^*b*^ (eV)	Relative d-manifold energy shift^*d*^ (eV)
		1^st^ peak	2^nd^ peak	Avg^*c*^	1^st^ peak	2^nd^ peak		
**1**	8332.9	2469.7	2470.4	2470.0	0.29	1.34	2472.1	0
**2**	8333.1	2469.9	2470.5	2470.3	0.47	1.15	2472.1	0.3
**4**	8332.7	2469.5	2470.2	2470.1	0.33	1.77	2472.2	0
[Ni(SPh) (PS_3_)]^–^	8333.0	2469.8	2470.4	2470.3	0.52	1.91	2472.3	0.1

^*a*^The peak energy is determined by the minimum of the second derivative.

^*b*^The peak energy and intensity is determined based on the spectral deconvolution.

^*c*^The intensity-weighted average energy is given here.

^*d*^Calculated from the difference of the thiolate peak energy and the intensity-weighted pre-edge peak energy.

**Fig. 4:**
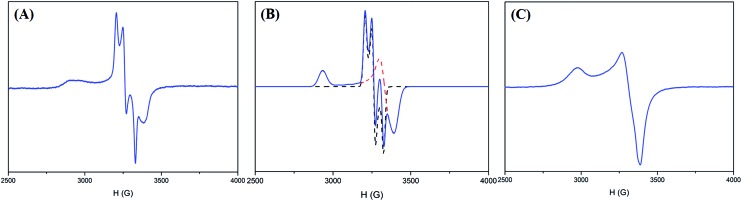
(A) EPR spectrum of complex **2** at 77 K, (B) simulated EPR spectrum (blue) of complex **2** combining [Ni^III^(L)(PS_3_)]^–^ (dashed red line) and the [CO_2_]˙^–^ radical (dashed black line), and (C) EPR spectrum of complex **4** at 77 K.

With regard to complex **4** as an isolobal equivalent to [Ni^III^:CO_2_], complex **2** is a Ni^III^ complex bearing a 17-valence-electron [CO_2_]˙^–^ ligand. The significantly lower intensity of the second S_1s_-to-Ni_3d_ transition peak observed in the S K-edge XAS spectrum of complex **2**, compared to that of complex **4**, discloses that the one extra electron shared by the axial Ni_3d_ orbital and 2π*u orbital of CO_2_ leads to a strengthening of the Ni^III^–CO_2_˙^–^ bond and stabilizes the coordination of κ^1^-[CO_2_]˙^–^ toward the Ni^III^ center (Fig. 3B and Table 1). As observed in complex [Ni^III^(L)(PS_3_)]^–^ (L = OMe, SEt, SPh), complex **4** displays an EPR silence at 300 K, an axial signal at *g* = 2.27 and 2.04 at 77 K, and an effective magnetic moment of 1.74 *μ*_B_ at 300 K (Fig. 4C and S7A†).^27^,^28^,^35^ The stabilization of the [CO_2_]˙^–^ radical through coordination to the Ni^III^ center in complex **2** was further evidenced by EPR spectroscopy.

As shown in Fig. 4A and B, the EPR spectrum of complex **2** at 77 K apparently resembles a combination of the typical EPR signal of [Ni^III^(L) (PS_3_)]^–^ (*g* = 2.31, 2.03, and 2.00) and the [CO_2_]˙^–^ radical with a contribution of Ni_3d_ leading to the observed *g* anisotropy (Fig. 4A and B).^36^ The spin quantitation of complex **2**, using complex **4** as a reference, demonstrates that the electronic structure of complex **2** is best described as a resonance hybrid between [Ni^III^:CO_2_˙^–^] and [Ni^II^:CO_2_], which is supported by the effective magnetic moment of 1.59 *μ*_B_ exhibited by complex **2** at 300 K (ESI Fig. S7B and S7C†).

The experimental valence-to-core X-ray emission (V2C XES) spectra of complexes **2** and **4** are presented in Fig. 3C. In comparison with complex **4**, the broad V2C transition peak of complex **2** at 8330.0 eV shifts from 8328.8 eV upon replacement of the [NCO]^–^ by the [CO_2_]˙^–^ ligand. DFT calculation was further pursued to verify the nature of the V2C transition(s). As shown in ESI Fig. S8A and S8B,† the DFT calculated V2C XES spectra resembles the experimental V2C features and, in particular, the trend of the energy shift comparing complexes **2** and **4**. The contribution of the 4σ_g_, 3σ_u_, and 1π_g_ orbitals of [NCO]^–^ and Ni_3d_–S_3p_ orbitals results in the V2C features of complex **4**.^37^ For complex **2**, the absence of transitions from the 3σ_u_ and 1π_g_ orbitals and an additional transition from the occupied 2π_u_ orbital of [CO_2_]˙^–^, in addition to the upward shift of the Ni_3d_–S_3p_ orbitals in complex **2**, rationalizes the higher V2C transition energy of complex **2** in comparison with complex **4**.

Complex [Ni(L)(P(C_6_H_3_-3-SiMe_3_-2-S)_3_)]^–^, embedded in a distorted trigonal bipyramidal geometry, features a wealth of chemical reactivity tailored by the oxidation state of Ni and coordinating ligand L (L = OPh, SPh, SePh and Cl for Ni^III^; L = CO, N_2_H_4_ for Ni^II^).^27^,^28^,^35^ To dissect the unique reactivity of [Ni^III^(OMe)(PS_3_)]^–^ (**1**) toward CO_2_ activation, the addition of CO_2_ into a THF solution of the representative Ni^III^-chalcogenate complex [Ni(SPh)(PS_3_)]^–^ was investigated. In contrast to the reaction of complex **1** and CO_2_ yielding complex **2**, complex [Ni^III^(SPh)(PS_3_)]^–^ is inert toward CO_2_. In addition, despite the potential reduction power of the Ni^II^ center in combination with the labile nature of the CO or N_2_H_4_ ligand, neither complex [Ni^II^(CO)(PS_3_)]^–^ nor complex [Ni^II^(N_2_H_4_)(PS_3_)]^–^ showed a reactivity toward CO_2_ when the THF solution of these Ni complexes was treated with CO_2_, respectively, at ambient temperature for 3 days. As shown in ESI Fig. S4† and Table 1, the covalent character of the [Ni^III^(SPh)] core, compared to the [Ni^III^(OMe)] core, derived from the σ-/π-electron-donating nature of the coordinated phenylthiolate ligand, rationalizes the inertness of [Ni^III^(SPh)(PS_3_)]^–^ toward CO_2_.^33^,^34^ Despite the labile nature of CO and N_2_H_4_, the inert reactivity of the Ni^II^ center toward CO_2_ demonstrates that the lowered Ni_3d_ manifold orbitals in Ni^III^ complex **1** attracts the binding of weak σ-donor CO_2_ and triggers the subsequent reduction of CO_2_ by the nucleophilic [OMe]^–^ in the immediate vicinity. The reactivity of complex **1** toward CO_2_, affording an O-bound [Ni^III^:CO_2_˙^–^] species, uncovers a novel strategy for the immobilization and reductive activation of CO_2_, contrary to the typical interaction of unoccupied CO_2_ 2π*u orbitals with filled high-lying metal d orbitals in low-valence metal complexes.^38^,^39^ Theoretically, lowering the energy of the 2π*u (6a_1_) (LUMO) orbital on CO_2_ for interaction with nickel orbitals binding by way of the O=C– unit may be responsible for the coordinated CO_2_ reduction and the nonlinearity of the triatomic CO_2_ molecule which contains 17 valence electrons, as reported by McGlynn and co-workers.^37^ These results illustrate aspects of how a coordinated ligand and the electronic state of the nickel center work in concert to trigger coordination and activation of CO_2_.

## Conclusions

Complex **1**, with the inherent combination of an electrophilic [Ni^III^(PS_3_)] core and a properly positioned [OMe]^–^ nucleophile, was employed to provide an optimum electronic condition to trap and activate CO_2_ to afford complex **2**, containing the O-coordinated [κ^1^-CO_2_]˙^–^ ligand. The Ni^III^-mediated reduction of CO_2_ by an adjacent [OMe]^–^ ligand immobilizes CO_2_ in the form of [Ni^III^:CO_2_˙^–^] and may open a novel CO_2_ activation pathway promoting the subsequent incorporation of CO_2_ in the buildup of functionalized products.

## Supplementary Material

Supplementary information

Crystal structure data
