# Supplementary material for: [NiIII(OMe)]-mediated reductive activation of CO2 affording a Ni(κ1-OCO) complex
Source: Chem Sci. 2016 Feb 24;7(6):3640–4. doi: 10.1039/c5sc04652a (PMC6008733; doi:10.1039/c5sc04652a)
Supplement: Supplementary file 1 [file SC-007-C5SC04652A-s001.pdf]

Electronic Supplementary Material (ESI) for Chemical Science.
This journal is © The Royal Society of Chemistry 2016

Supplementary Information

[Ni^{III}(OMe)]-Mediated Reductive Activation of CO_2 Affording a Ni(κ^1-OCO) Complex

Tzung-Wen Chiou,[1*] Yen-Ming Tseng,[1] Tsai-Te Lu,[2] Tsu-Chien Weng,[3] Dimosthenes Sokaras,[3] Wei-Chieh Ho,[1] Ting-Shen Kuo,[4] Ling-Yun Jang,[5] Jyh-Fu Lee,[5] Wen-Feng Liaw[1*]

Affiliations:

[1]*Department of Chemistry, National Tsing Hua University, Hsinchu, 30013, Taiwan*

[2]*Department of Chemistry, Chung Yuan Christian University, Chungli, 32023, Taiwan*

[3]*SLAC National Accelerator Laboratory 2575 Sand Hill Rd. Menlo Park, CA 94025, USA*

[4]*Department of Chemistry, National Taiwan Normal University, Taipei, 10610, Taiwan*

[5]*National Synchrotron Radiation Research Center, Hsinchu, 30013, Taiwan.*

**To whom correspondence should be addressed*

E-mail: d9623817@oz.nthu.edu.tw (T.-W.C.); wfliaw@mx.nthu.edu.tw (W.-F.L)

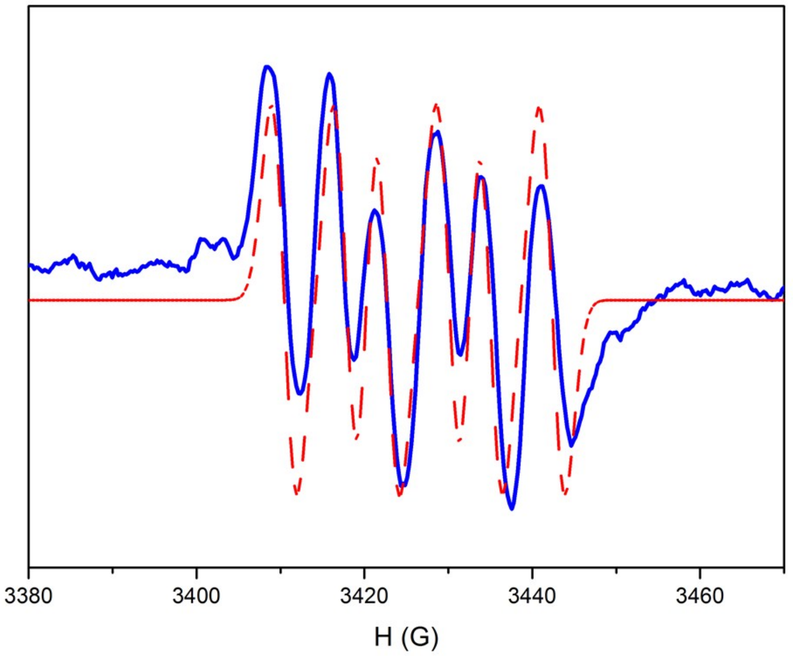

Figure S1. EPR spectrum (blue) of the THF-diethyl ether solution obtained from reaction of complex **1** and CO_2 in the presence of DMPO. Simulated EPR spectrum (red dashed line) using g = 2.0055, A_N = 12.25 G, and A_H = 7.40 G indicates the formation of methoxy radical trapped by DMPO.

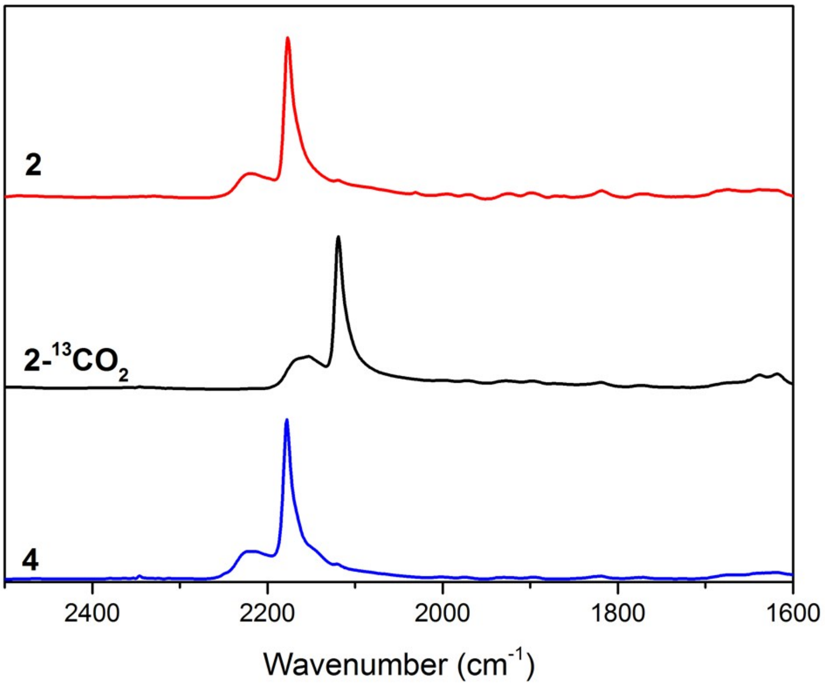

Figure S2. IR spectra of complexes **2** (red), **2-^{13}CO$_2$** (black), and **4** (blue). An isotopic shift of IR ν_{OCO} stretching peak from 2177 cm^{-1} to 2117 cm^{-1} (KBr) was observed in the $^{13}CO_2$ labeling experiment. An IR ν_{NCO} stretching peak at 2178 cm^{-1} (KBr) was observed in complex **4**.

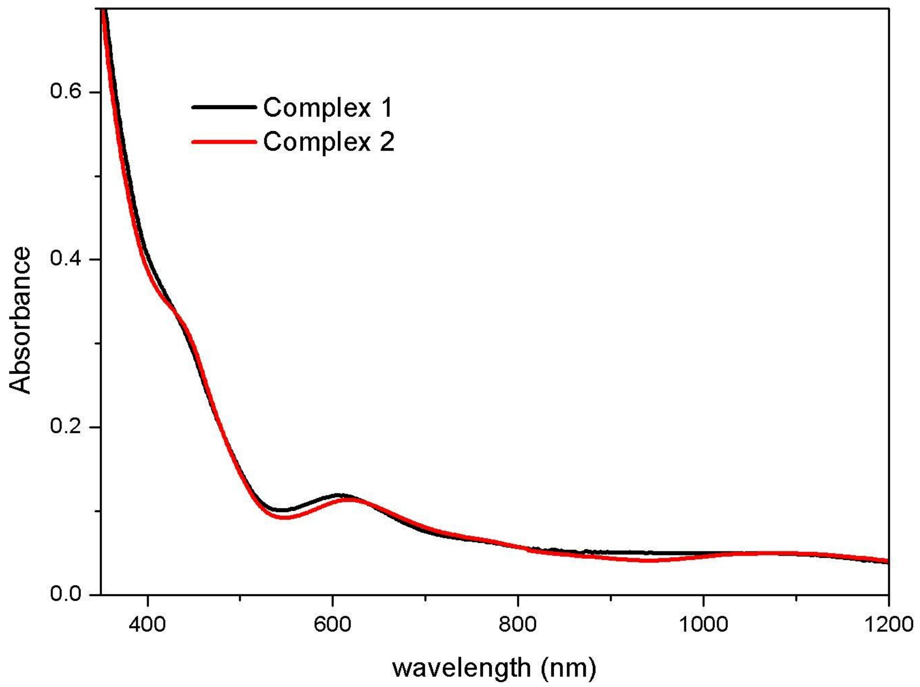

Figure S3. UV-vis spectra of transformation of complex **1** to **2** in THF. The intense bands at 419 and 605 nm (black) exhibited by complex **1** disappeared with simultaneous formation of absorption bands at 425 and 610 nm (red) indicating the formation of complex **2**.

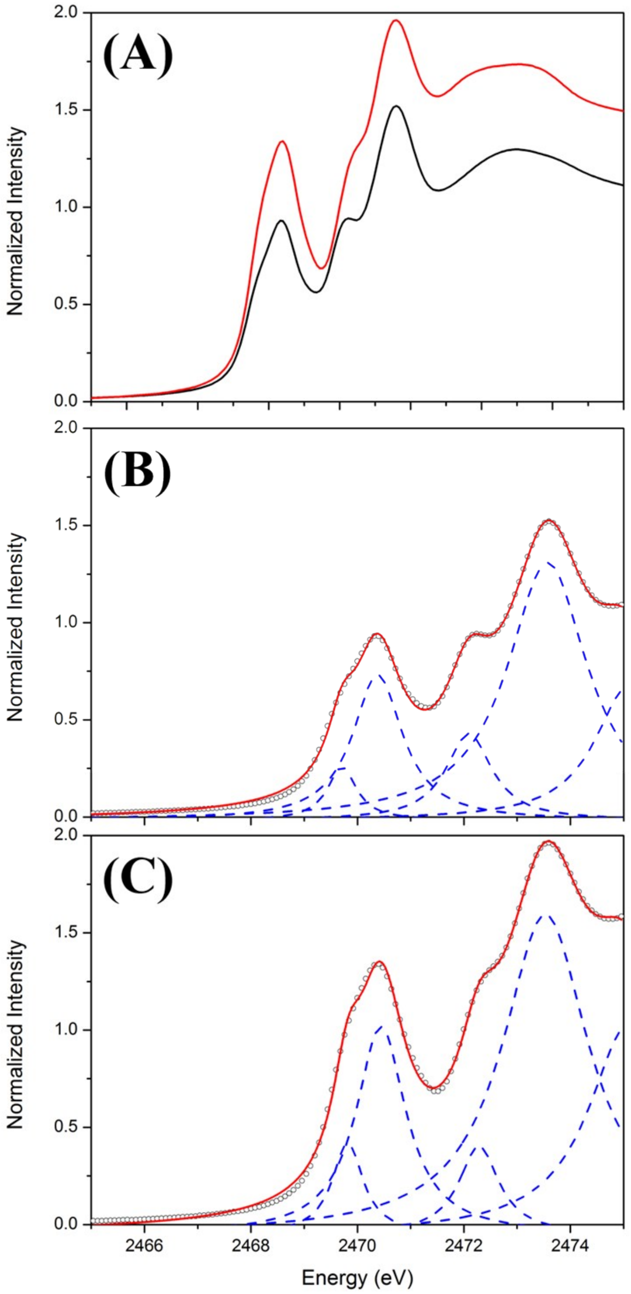

Figure S4. (**A**) S K-edge XAS of complexes **1** (black) and $[Ni^{III}(SPh)(PS_3)]^-$ (red). Spectral deconvolution of **(B)** complex **1** and **(C)** complex $[Ni^{III}(SPh)(PS_3)]^-$. Spectral deconvolution of the data are shown as blue dashed lines, whereas red line indicates the accumulation of each transitions. As shown in Table 1, complex **1** exhibits a pre-edge absorption intensity of 1.63 derived from $S_{1s} \rightarrow Ni_{3d}$ transition, while a pre-edge absorption intensity of 2.43 is displayed by complex $[Ni^{III}(SPh)(PS_3)]^-$. The higher S K-edge pre-edge absorption intensity observed in complex $[Ni^{III}(SPh)(PS_3)]^-$ delineates that the σ-/π-electron-donating nature of the coordinated phenylthiolate ligand, presumably, builds a more covalent Ni(III) center in complex $[Ni^{III}(SPh)(PS_3)]^-$ compared to complex **1**.

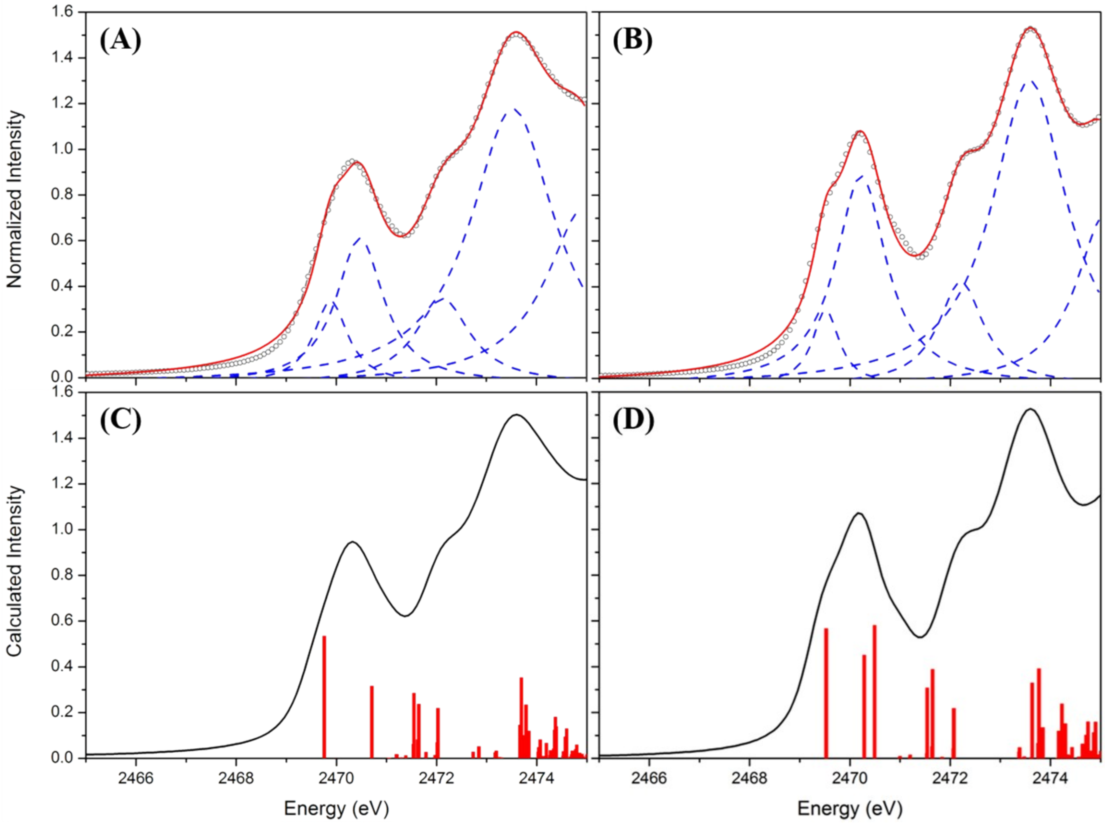

Figure S5. Experimental S K-edge XAS (open circle) of (**A**) complex **2** and (**B**) complex **4**. DFT calculated S K-edge X-ray absorption peaks (vertical lines) of (**C**) complex **2** and (**D**) complex **4** with experimental S K-edge XAS shown in black line. Spectral deconvolution of the data is shown as dashed lines, whereas red line indicates the accumulation of each transitions.

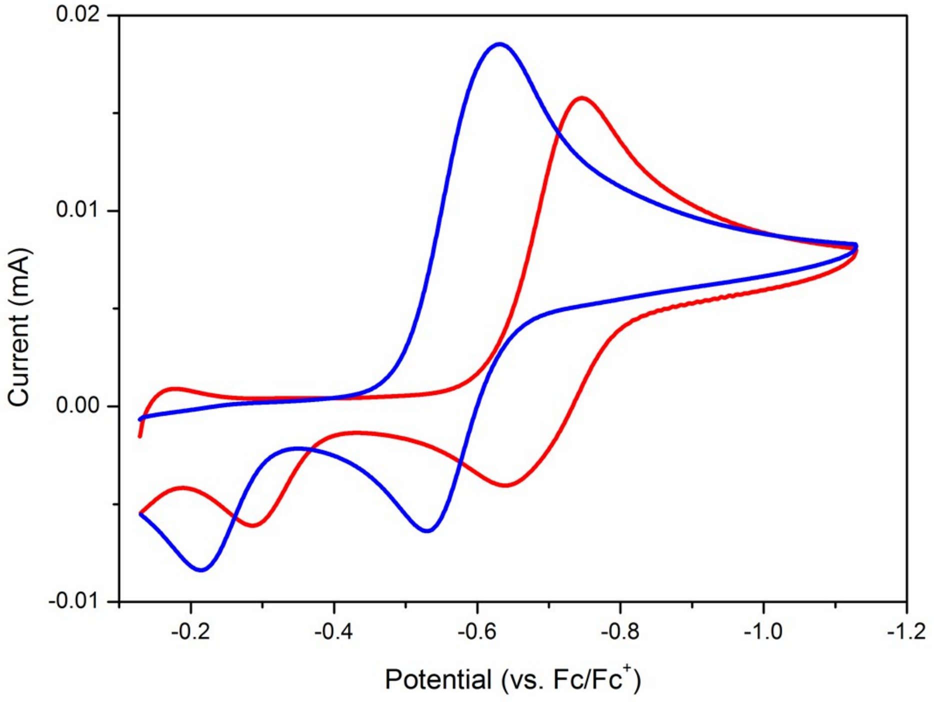

Figure S6. Cyclic voltammogram of 2 mM solution of complex **2** (red) and complex **4** (blue) in CH_3CN with 0.1 M of $[nBu_4N][PF_6]$ electrolyte. Scan rate: 50 mVs^{-1}.

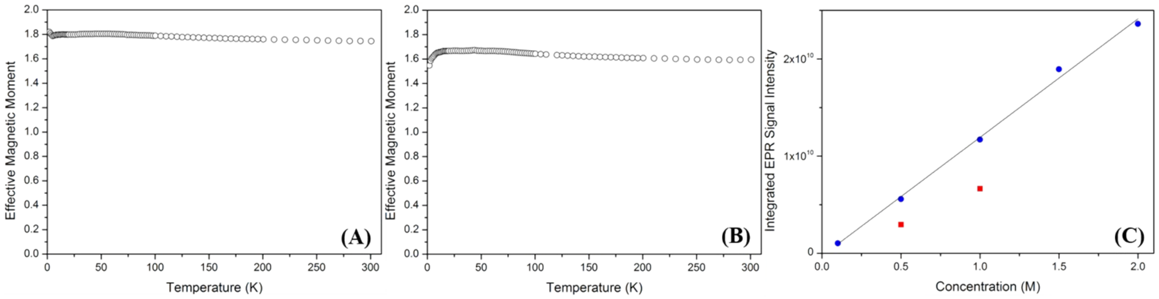

Figure S7. (**A**) Plot of effective magnetic moment vs. temperature (2-300 K) for complex **4**. (**B**) Plot of effective magnetic moment vs. temperature (2-300 K) for complex **2**. (**C**) Intensity for the double integration of EPR signal intensity of 0.1, 0.5, 1, 1.5, and 2 M of complex **4** (blue dot) and 0.5 and 1 M of complex **2** (red square) in THF.

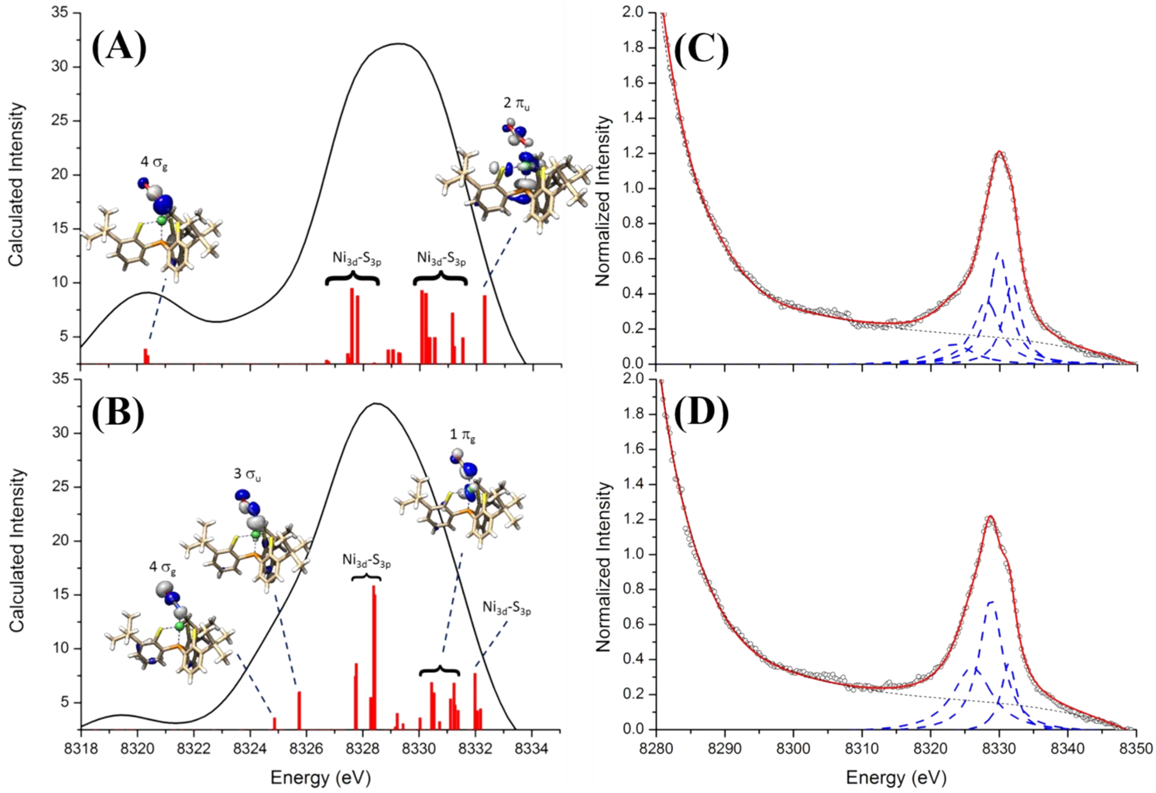

Figure S8. DFT calculated V2C XES spectra of (**A**) complex **2** and (**B**) complex **4**, and the corresponding molecular orbitals for each of the transitions. Spectral deconvolution of V2C XES spectra of (**C**) complex **4** and (**D**) complex **2**. 16-valence-electron triatomic molecule $[NCO]^{-}$ (or OCO) contains one $3\sigma_g$ (N_{2s}-C_{2s}-O_{2s}), one $2\sigma_u$ (N_{2s}-O_{2s}^{*}), one $4\sigma_g$ (N_{2s}-C_{2s}-O_{2s}^{*}), two $1\pi_u$ (N_{2p}-C_{2p}-O_{2p}), one $3\sigma_u$ (N_{2p}-C_{2p}-O_{2p}), two $1\pi_g$ (N_{2p}-O_{2p}^{*}) occupied molecular orbitals. One-electron reduction of CO_2 leads to the 17-valence-electron $[CO_2^{\bullet-}]$ containing a half-filled $2\pi_u$ (O_{2p}-C_{2p}-O_{2p}^{*}) molecular orbitals. In Ni V2C XES of complex **4**, mixing of $4\sigma_g$, $3\sigma_u$, $1\pi_g$ orbitals of $[NCO]^{-}$ with Ni_{4p} results in the V2C transitions, in addition to the contribution from Ni_{3d}-S_{3p} molecular orbitals. Compared to the Ni-NCO bond distance of 1.933 Å in complex **4**, the longer Ni-OCO bond distance of 2.028 Å in complex **2** diminished the interaction between $3\sigma_u/1\pi_g$ orbitals of CO_2 and Ni_{4p} and explains the absent contribution of $3\sigma_u/1\pi_g$ orbitals of OCO to the V2C feature. The delocalization nature of $2\pi_u$ orbitals of CO_2, however, retains its the bonding interaction with Ni(III) center, as revealed by S K-edge XAS, and leads to the V2C transition at higher energy. The bent O–C–O bond angle of 171.7°, presumably, rationalizes the shift of $4\sigma_g$ orbitals of CO_2 in complex **2** to lower energy. Calculated peaks are displayed in the vertical lines. Spectral deconvolution of the data is shown as blue dashed lines.

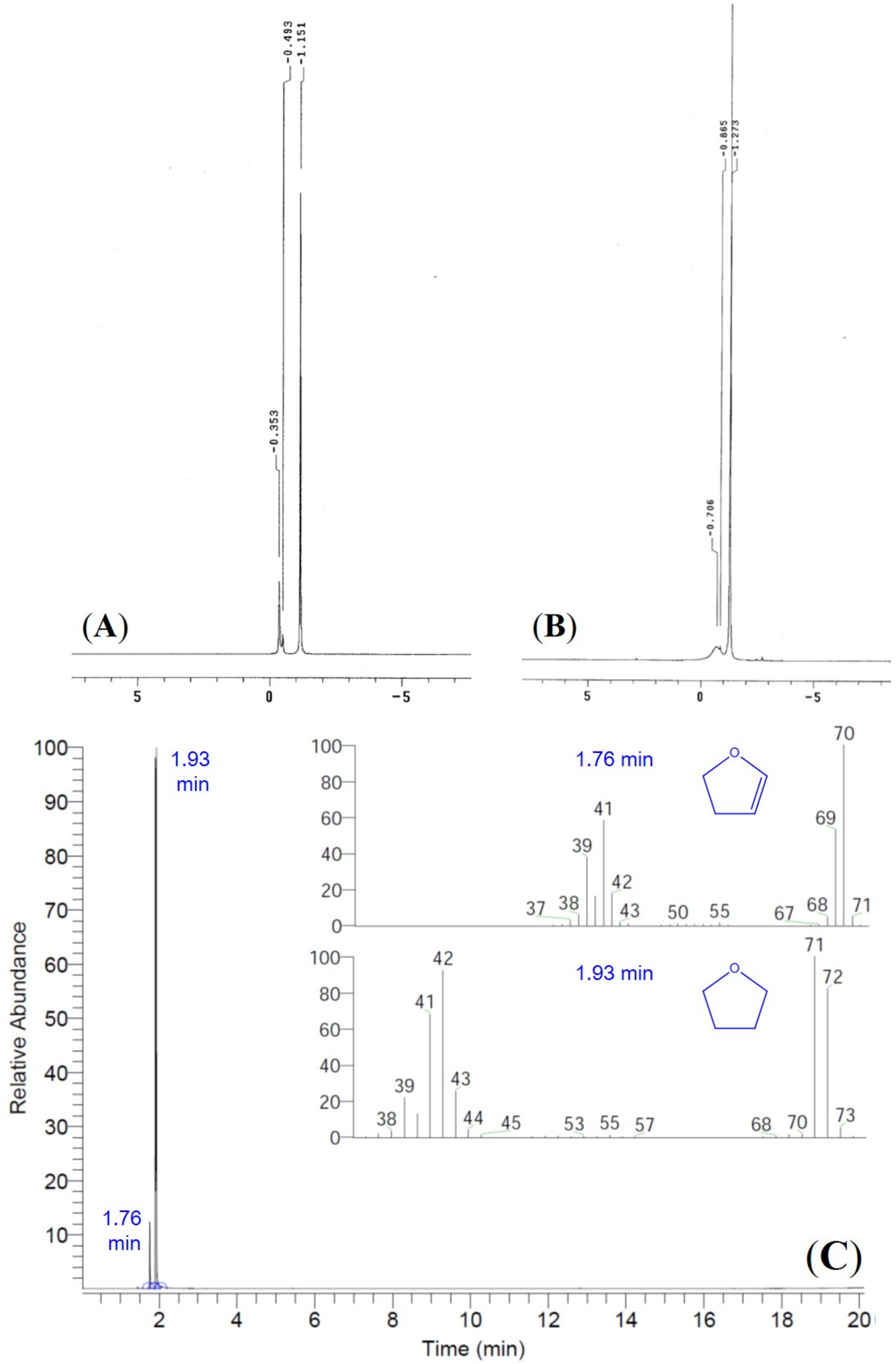

Figure S9. ^{2}H-NMR spectra of (**A**) CD_3OD in THF and (**B**) reaction solution of complex [PPN][Ni(OCD_3)(PS_3)] and CO_2 in THF. D_2O was used as an external reference. (**C**) GC chromatogram for the reaction solution of complex [PPN][Ni(OCD_3)(PS_3)] and CO_2 in THF, whereas the corresponding mass spectra for the peaks at 1.76 min (2,3-dihydrofuran) and 1.93 min (THF) are shown in the inset.

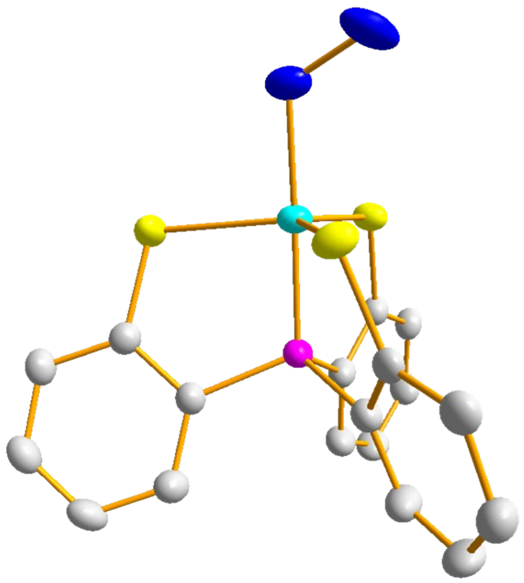

Figure S10. ORTEP drawing scheme of complex $[Ni(N_2H_4)(PS_3)]^-$ with thermal ellipsoids drawn at 50% probability level. The Ni, P, S, N, and C atoms are shown as light blue, purple, yellow, blue, and white ellipsoids. H atom and TMS group are omitted for clarity. Selected bond distances (Å) and angles (°) for complex $[PPN][Ni(N_2H_4)(PS_3)]$: Ni–N 1.980(2); Ni–P 2.078(1); Ni–S 2.288(1), 2.265(1), and 2.375(1); N–N 1.439(4); N–Ni–P 176.8(7); Ni–N–N 116.8(2).

Methods

Manipulations, reactions, and transfers were conducted under N_2 according to Schlenk techniques or in a glovebox. Solvents were distilled under N_2 from appropriate drying agents (acetonitrile from CaH_2-P_2O_5; methylene chloride from CaH_2; diethyl ether, hexane and THF from sodium benzophenone) and stored in dried, N_2-filled flasks over 4 Å molecular sieves. N_2 was purged through these solvents before use. Solvent was transferred to the reaction vessel via a stainless cannula under a positive pressure of N_2. The reagents bis(triphenylphosphoranylidene)ammonium chloride ([PPN][Cl]; Fluka), [Na][OC_6H_5], [*n*-Bu_4N][OCH_3] (Fluka), 5,5-Dimethyl-1-pyrroline *N*-oxide (DMPO; AK Scientific inc.), nickel(II) dichloride, CD_3OD (Aldrich), and N_2H_4•H_2O (80% in H_2O; SHOWA) were used as received. Compounds [PPN][Ni(OCH_3)(PS_3)] (PS_3 = P(*o*-C_6H_3-3-$SiMe_3$-2-S)$_3$), [PPN][Ni(Cl)(PS_3)], [PPN][Ni(SPh)(PS_3)], [PPN][Ni(CO)(PS_3)], and [Ni(PS_3)]$_2$ were synthesized by published procedures.[1-3] Infrared spectra of the ν_{OCO} stretching frequencies were recorded on a PerkinElmer model spectrum One B spectrometer with KBr. UV-vis spectra were recorded on a Jasco V-570 spectrometer. Analyses of C, H, and N were obtained with a CHN analyzer (Heraeus). ^2H NMR spectra were acquired on a VARIAN UNITY INOVA 500 NMR spectrometer. GC-MS spectra were obtained with a AccuTOF GCX (JEOL). All electrochemical measurements were performed in a three-electrode cell with a CHI model 621b potentiostat (CH Instrument) instrumentation. Cyclic voltammograms in CH_3CN were obtained from 2 mM analyte concentration in O_2-free MeCN using 0.1 M [n-Bu_4N][PF_6] as the supporting electrolyte. The potential was measured at 298 K vs Ag/$AgNO_3$ reference electrode (0.01 M $AgNO_3$) by using a glassy carbon working electrode (surface area = 0.0707 cm^2) and a platinum wire auxiliary electrode at a scan rate of 50 mV/s. The potentials are reported against the ferrocenium/ferrocene (Fc^+/Fc) couple.

Preparation of [PPN][Ni(κ[1]-OCO)(PS$_3$)] (2), PS$_3$ = P(*o*-C_6H_3-3-SiMe$_3$-2-S)$_3$. The thermally stable complex [PPN][Ni(OCH$_3$)(PS$_3$)] (**1**) (0.121 g, 0.1 mmol) in THF (5 mL) was purged by pure CO_2 gas (1 atm) at 298 K for 30 min. The reaction solution was then stirred under CO_2 atmosphere at ambient temperature overnight. The solution color changing from blue green to yellow green was observed. Shift of UV-vis absorption bands from 419, 605 nm to 425, 610 nm implicates the formation of [PPN][Ni(κ[1]-OCO)(PS$_3$)] (**2**). The mixture solution was then filtered through Celite to remove the insoluble unidentified solid. Diethyl ether (20 mL) was added to precipitate the dark-green solid characterized as complex **2** (yield 0.085 g, 70%). The THF solution of complex **2** layered with diethyl ether at ambient temperature for 5 days led to the formation of dark green needle crystals of complex **2** suitable for X-ray crystallography. Complex **2**: IR $\nu(^{12}CO_2)$: 2177 cm^{-1} (KBr); 2226 s cm^{-1} (THF); $\nu(^{13}CO_2)$: 2117 cm^{-1} (KBr). Absorption spectrum (THF) [λ_{max}, nm (ε, $M^{-1}cm^{-1}$)]: 425 (6610), 610 (2370), 734 (1400), 1031 (1014). Anal. Calcd for $C_{64}H_{66}P_3NiNO_2S_3Si_3$: C, 63.36; H, 5.48; N, 1.15. Found: C, 64.01; H, 5.75; N, 0.93.

To verify the accompanied formation of methoxy radical in the reaction of complex **1** and CO_2, THF solution (5 mL) of complex **1** (0.121 g, 0.1 mmol) was purged with $CO_{2(g)}$ for 5 min in the presence of DMPO (0.012 g, 0.1 mmol). After the reaction solution was stirred for another 30 min, diethyl ether (30 mL) was added to precipitate complex **2** (yield 0.098 g, 81%). The THF-diethyl ether solution was then transferred to EPR tube for EPR experiment. The EPR signal at g = 2.0055 with A_N = 12.25 G and A_H = 7.40 G indicates the formation of methoxy radical trapped by DMPO.

To characterize the fate of the methoxy radical, complex **1** containing a deuterated methoxide ligand, [PPN][Ni(OCD$_3$)(PS$_3$)], was prepared. Reaction of $CO_{2(g)}$ with complex [PPN][Ni(OCD$_3$)(PS$_3$)] resulted in the formation of complex **2** in a similar

fashion described above. In addition, accompanied formation of CD_3OH and 2,3-dihydrofuran, as verified by ^{2}H-NMR and GC-MS (Figure S9), lends an alternative support to the transient formation of methoxy radical, which abstracts the hydrogen atom from the reaction solvent THF and affords CD_3OH and 2,3-dihydrofuran.

Reaction of Complex 2 and $CO_{(g)}$. To a 5-mL THF solution of complex **2** (0.125 g, 0.1 mmol) was injected with 5 mL of $CO_{(g)}$. After the reaction solution was stirred for 1 day, appearance of IR ν_{CO} peak at 2027 cm^{-1} indicated the formation of the reported complex [PPN][Ni(CO)(PS_3)].[1-3] The gas collected from the headspace of this reaction tube was then injected to gas chromatography (SRI 8610C). A peak with retention time of 30.68 min observed in the GC chromatogram indicated the release of $CO_{2(g)}$ during the transformation of complex **2** to complex $[Ni^{II}(CO)(PS_3)]^-$.

Preparation of [PPN][Ni(NCO)(PS_3)] (4). The mixed solvent (THF-CH_3CN volume ratio 6:2 (mL)) was added into complexes [PPN][Ni(Cl)(PS_3)] (**3**) (0.126 g, 0.1 mmol) and [K][NCO] (0.008 g, 0.1 mmol) at 298 K and then the reaction solution was stirred overnight at ambient temperature. The solution color changed from yellow green to blue green. UV-vis absorption bands shifted from 430, 620 nm to 430, 604 nm and implicated the formation of [PPN][Ni(NCO)(PS_3)] (**4**). The mixture solution was then filtered through Celite to remove the insoluble [K][Cl] solid. Diethyl ether (20 mL) was added to precipitate the dark green solid characterized as complex **4** (yield 0.103 g, 85%). Since complex **4** is slightly soluble in THF, the THF-CH_3CN mixed solution of complex **4** is prepared for the crystal growth. The THF-CH_3CN mixed solution of complex **4** layered with diethyl ether at ambient temperature for 3 days led to the formation of $[Ni^{III}(NCO)(PS_3)]^-$ (**4**) crystals (dark green needle) suitable for X-ray crystallography. Complex **4**: IR ν(NCO): 2178 cm^{-1} (KBr). Absorption spectrum (CH_3CN) [λ_{max}, nm (ε,

$M^{-1}cm^{-1}$)]: 430 (5664), 604 (2206), 729 (1452), 998 (907). Anal. Calcd for $C_{64}H_{66}P_3NiN_2OS_3Si_3$: C, 63.46; H, 5.49; N, 2.31. Found: C, 63.80; H, 5.69; N, 2.36.

Preparation of [PPN][Ni(N_2H_4)(PS_3)]. A 5-mL THF-CH_3CN solution (volume ratio 3:1) of complex [PPN][Ni(Cl)(PS_3)] (0.242 g, 0.2 mmol) in a 20 mL Schlenk tube was added 0.12 mL of N_2H_4•$H_2O_{(aq)}$ (80%) under positive N_2. The mixture solution was stirred for 1 h and then filtered through Celite to remove the insoluble solid in anaerobic condition. Diethyl-ether was then added to the filtrate (THF-CH_3CN solution) leading to the precipitation of the red-brown solid [PPN][Ni(N_2H_4)(PS_3)] (yield 0.168 g, 70%). The THF-CH_3CN solution (volume ratio 3:1) of complex [PPN][Ni(N_2H_4)(PS_3)] layered with diethyl ether at ambient temperature for 3 days led to the formation of red-brown needle crystals of complex [PPN][Ni(N_2H_4)(PS_3)] suitable for X-ray crystallography (SI Figure S10). Absorption spectrum (THF) [λ_{max}, nm (ε, $M^{-1}cm^{-1}$)]: 780 (436). Anal. Calcd for $C_{67}H_{80}N_4NiOP_3S_3Si_3$•1/2$N_2H_4$: C, 62.41; H, 6.25; N, 4.35. Found: C, 62.19; H, 6.36; N, 4.85.

Reaction of complex [PPN][Ni(L)(PS_3)] (L = SPh, N_2H_4, and CO) and CO_2. THF solution (5 mL) of complex [PPN][Ni(N_2H_4)(PS_3)] (0.132 g, 0.2 mmol) was purged by pure CO_2 gas (1 atm) at 298 K for 30 min. The reaction was monitored by UV-vis spectrometry. No change of the UV-vis absorption band at 780 nm after the reaction solution was stirred under CO_2 atmosphere at ambient temperature for 3 days indicates the inertness of complex [PPN][Ni(N_2H_4)(PS_3)] toward CO_2. The inertness of complex [PPN][Ni(SPh)(PS_3)] as well as complex [PPN][Ni(CO)(PS_3)] toward $CO_{2(g)}$ was verified in a similar fashion.

EPR Spectroscopy. EPR measurements were performed at X-band using a Bruker EMX spectrometer equipped with a Bruker TE102 cavity. The microwave frequency was

measured with a Hewlett-Packard 5246L electronic counter. X-band EPR spectra of complexes **2** and **4** in THF and THF-CH_3CN were obtained with a microwave power of 1.5 and 1.5 mW, modulation amplitude of 1.60 and 1.60 G at 100 KHz, and microwave frequency at 9.459 and 9.469 GHz, respectively. X-band EPR spectrum of the THF-diethyl ether solution obtained from reaction of complex **1** and CO_2 in the presence of DMPO was collected with a microwave power of 15.0 mW, modulation amplitude of 1.60 G at 100 KHz, and microwave frequency at 9.618 GHz. The spectral simulation was performed using Bruker WinEPR.

Magnetic Measurements. The magnetization data were recorded on a SQUID magnetometer (MPMS5 Quantum Design company) with an external 0.5 T magnetic field for complexes **2** and **4** in the temperature range 2-300 K. The magnetic susceptibility of the experimental data was corrected for diamagnetism by the tabulated Pascal's constants.

Crystallography. Crystallographic data of complexes **2** (CCDC 785531), **4** (CCDC 1435237) and [PPN][Ni(N_2H_4)(PS_3)] (CCDC 1435238) were deposited in Cambridge Crystallographic Data Centre. The crystals chosen for X-ray diffraction studies measured 0.30 × 0.15 × 0.12 mm for complex **2**, 0.25 × 0.25 × 0.18 mm for complex **4**, and 0.34 × 0.28 × 0.22 mm for [PPN][Ni(N_2H_4)(PS_3)], respectively. Each crystal was mounted on a glass fiber and quickly coated in epoxy resin. Unit-cell parameters were obtained by least-squares refinement. Diffraction measurements for complexes **2**, **4** and [PPN][Ni(N_2H_4)(PS_3)] were carried out on a SMART CCD (Nonius Kappa CCD) diffractometer with graphite-monochromated Mo K_α radiation (λ = 0.7107 Å) and between 1.46 and 26.45° for complex **2**, between 1.46 and 26.39° for complex **4**. Least-squares refinement of the positional and anisotropic thermal parameters of all non-hydrogen atoms and fixed hydrogen atoms was based on F^2. A SADABS absorption correction was made.[4] The SHELXTL structure refinement program was employed.[5] It is

noted that the crystal system, space group and unit cell dimensions of complexes **2**, **4** and the published [PPN][Ni(CH_2CN)(PS_3)] are all similar (Tables S1).[2] These results implicated that the [Ni^{III}(PS_3)] complexes axially coordinated by the linear three-atomic molecules have similar crystalline packing.

X-ray Absorption Measurements. All X-ray absorption experiments were carried out at the National Synchrotron Radiation Research Center (NSRRC), Hsinchu, Taiwan. Both Ni and S K-edge XAS was recorded at room temperature. For Ni K-edge measurements, the experiments were performed in transmission mode at the BL-17C X-ray Wiggler beamline with a double crystal Si(111) monochromator. Samples of complexes **1**, **2**, and **4** were ground to powder from single crystals, and sealed into Al spacers with Kapton tape under dry N_2 atmosphere in a glove box. The spectra were scanned from 8.132 to 9.326 KeV using a gas-ionization detector. A reference Ni foil is always used simultaneously for the calibration of energy. The ion chambers used to measure the incident (I_0) and transmitted (I) intensities were filled with a mixture of N_2 and He gases and a mixture of N_2 and Ar gases, respectively.

The S K-edge data were collected in fluorescence mode at BL-16A with a Si(111) monochromator. The energy resolution $\Delta E/E$ is 1.4 x 10^{-4}. The energy is scanned from 2.42 to 2.77 keV. A Lytle detector was employed for fluorescence measurements in which the sample chamber is filled with high-purity He gas to avoid air absorption. Samples of complexes **1**, **2**, **4**, and [PPN][Ni(SPh)(PS_3)] were ground to powder from single crystals and secured in the bag made of 3.6-μm Mylar film. The absorption of the S K-edge region of the empty bag made of 3.6-μm Mylar film was verified to be negligible. The photon energy was calibrated to the maximum of the first pre-edge feature of $Na_2S_2O_3.5H_2O$ at 2472.02 eV. The pre-edge features at the S K-edge were fitted using the

software package ORIGIN and modeled by Lorentzian functions over the range of 2465-2475 eV.

XES measurement. All spectra were collected at beamline BL 6-2 of SLAC National Accelerator Laboratory in Menlo Park, U.S.A. The bare Si surface lane of a white beam mirror before the double-crystal monochromator Si(311) was used to reject the high order harmonics. The X-ray energy was calibrated with Ni foil with the first inflection point assigned to 8331.6 eV. X-ray beam was focused with Kirkpatric-Baez mirrors at the sample with a spot size of 0.5 mm (fwhm, H) by 0.1 mm (fwhm, V). The full photon flux on the sample was 2×10^{13} ph/s at 8600 eV.

Ni X-ray emission spectra were collected with incident X-ray energy set at 8600 eV. Five 1-m spherically-bent crystal analyzer Ge(620), sitting at 90-degree to the X-ray beam, were used to resolve fluorescence, with an energy bandwidth of 1.0 eV, and focused to the detector, avalanche photo-diode, sitting above the sample in a vertical Rowland geometry. A helium-filled bag with Kapton windows (25 μm thickness) was placed in between the sample, the analyzers, and the detector to minimize attenuation of the fluorescence signal due to the air absorption.

Radiation-damage assessment was performed on each sample before XES measurement to determine the X-ray attenuation power (either 40 or 60 μm Al depending on samples) and the corresponded exposure time on one fresh spot. Each XES spectrum was collected on multiple fresh spots in order to avoid X-ray induced photo-reduction.

X-ray emission spectra were collected from 8235 to 8290 eV for Ni K_β region, and from 8285 to 8350 eV for Ni K-valence region, with an increment of 0.25 eV and a counting time of 1 sec at each point. For each samples, 25 K_β scans and 180 K-valence scans were merged, normalized to the incident photon flux, and further normalized by the

total integrated area of the full spectra. For each normalized spectra, the background tail from the K_β main line and the valence-to-core transitions were fit with Lorentzian functions using the software package ORIGIN.

DFT calculation. S K-edge calculation was conducted based on the reported procedure.[6] The B3LYP exchange functional and def2-TZVP(-f) basis set on the Ni, S, N, O, C, and H atoms were used for electronic structure calculation. X-ray crystallographic structure of complexes **2** and **4** were used in the calculation. For Ni K-valence XES, one-electron DFT calculations were carried out using quantum chemistry program ORCA 3.0.[7] The exchange-correlation functionals BP86 was used in the calculations with the CP(PPP) basis set on Ni (with a special integration accuracy of 7) and the TZVP basis set on all remaining atoms. Ni K-valence XES spectra were simulated with dipole transitions between 1s and valence Kohn-Sham orbitals without core-hole relaxation as reported. Simulated Fe K-valence spectra were broadened with a Gaussian width of 2.5 eV and offset by +213.8 eV from the K_β main peak alignment. Illustrations of the molecular orbitals were generated using the Chimera 1.8.1 program with the iso-surface value set at ±0.045 au.

References

1. Lee, C.-M., Chen, C.-H., Ke, S.-C., Lee, G.-H. & Liaw, W.-F. Mononuclear nickel(III) and nickel(II) thiolate complexes with intramolecular S-H proton interacting with both sulfur and nickel: Relevance to the [NiFe]/[NiFeSe] hydrogenases. *J. Am. Chem. Soc.* **126**, 8406-8412 (2004).
2. Lee, C.-M., Chuang, Y.-L., Chiang, C.-Y., Lee, G.-H. & Liaw, W.-F. Mononuclear Ni(III) complexes $[Ni^{III}(L)(P(C_6H_3\text{-}3\text{-}SiMe_3\text{-}2\text{-}S)_3)]^{0/1-}$ (L = thiolate, selenolate, CH_2CN, Cl, PPh_3): Relevance to the nickel site of [NiFe] hydrogenases. *Inorg. Chem.* **45**, 10895-10904 (2006).
3. Chiou, T.-W. & Liaw, W.-F. Mononuclear nickel(III) complexes $[Ni^{III}(OR)(P(C_6H_3\text{-}3\text{-}SiMe_3\text{-}2\text{-}S)_3)]^-$ (R = Me, Ph) containing the terminal alkoxide ligand: Relevance to the nickel site of oxidized-form [NiFe] hydrogenases. *Inorg. Chem.* **47**, 7908-7913 (2008).
4. Sheldrick, G. M. *SADABS, Siemens Area Detector Absorption Correction Program*; University of Göttingen: Germany, 1996.

5. Sheldrick, G. M. *SHELXTL, Program for Crystal Structure Determination;* Siemens Analytical X-ray Instruments Inc.: Madison, WI, 1994.

6. T.-T. Lu, S.-H. Lai, Y.-W. Li, I-J. Hsu, L.-Y. Jang, J.-F. Lee, I-C. Chen and W.-F. Liaw, *Inorg. Chem.*, 2011, **50**, 5396.

7. a) U. Bergmann, J. Bendix, P. Glatzel, H. B. Gray, S. P. Cramer, *J. Chem. Phys.* **2002**, *116*, 2011-2015; b) B. Lassalle-Kaiser, T. T. Boron, 3rd, V. Krewald, J. Kern, M. A. Beckwith, M. U. Delgado-Jaime, H. Schroeder, R. Alonso-Mori, D. Nordlund, T. C. Weng, D. Sokaras, F. Neese, U. Bergmann, V. K. Yachandra, S. DeBeer, V. L. Pecoraro, J. Yano, *Inorg. Chem.* **2013**, *52*, 12915-12922; c) F. Neese, *WIRES Comput. Mol. Sci.* **2012**, *2*, 73-78.

Table S1. Comparisons of crystal system, space group, unit cell dimensions and symmetry operate of complexes **2**, **4** and [PPN][Ni(CH_2CN)(PS_3)].[2]

	Complex **2**	Complex **4**	Complex [PPN][Ni(CH_2CN)(PS_3)]
CCDC number	785531	1435237	636509
Empirical formula	C68 H74 N Ni O3 P3 S3 Si3	C68 H74 N2 Ni O2 P3 S3 Si3	C69 H76 N2 Ni O P3 S3 Si3
Formula weight	1285.35	1283.36	1281.39
Temperature	100(2) K	100(2) K	150(1) K
Wavelength	0.71073 Å	0.71073 Å	0.71073 Å
Crystal system	Orthorhombic	Orthorhombic	Orthorhombic
Space group	*Pbca*	*Pbca*	*Pbca*
Unit cell dimensions	a = 27.4702(8) Å, α= 90°	a = 27.5733(7) Å, α= 90°	a =27.6146(4) Å, α= 90°
	b = 17.1432(6) Å, β= 90°	b = 17.0409(5) Å, β= 90°	b =17.3570(2) Å, β= 90°
	c = 27.9778(8) Å, γ = 90°	c = 27.8585(8) Å, γ= 90°	c =27.9427(4) Å, γ= 90°
Volume	13175.5(7) $Å^3$	13090.0(6) $Å^3$	13393.1(3) $Å^3$
Z	8	8	8
Density (calculated)	1.296 Mg/m^3	1.302 Mg/m^3	1.271 Mg/m^3
Absorption coefficient	0.563 mm^{-1}	0.566 mm^{-1}	0.552 mm^{-1}
Goodness-of-fit on F^2	1.042	1.171	1.007
Final R indices [I>2sigma(I)]	R1 = 0.0552, wR2 = 0.1449	R1 = 0.0382, wR2 = 0.1060	R1 =0.0528, wR2 = 0.1090
R indices (all data)	R1 = 0.0912, wR2 = 0.1725	R1 = 0.0499, wR2 = 0.1276	R1 =0.1242, wR2 = 0.1468

Complex **2**

_symmetry_equiv_pos_as_xyz
'x, y, z'
'-x+1/2, -y, z+1/2'
'-x, y+1/2, -z+1/2'
'x+1/2, -y+1/2, -z'
'-x, -y, -z'
'x-1/2, y, -z-1/2'
'x, -y-1/2, z-1/2'
'-x-1/2, y-1/2, z'

Complex [PPN][Ni(CH_2CN)(PS_3)]

_symmetry_equiv_pos_as_xyz
'x, y, z'
'-x+1/2, -y, z+1/2'
'-x, y+1/2, -z+1/2'
'x+1/2, -y+1/2, -z'
'-x, -y, -z'
'x-1/2, y, -z-1/2'
'x, -y-1/2, z-1/2'
'-x-1/2, y-1/2, z'

Complex **4**

_symmetry_equiv_pos_as_xyz
'x, y, z'
'-x+1/2, -y, z+1/2'
'-x, y+1/2, -z+1/2'
'x+1/2, -y+1/2, -z'
'-x, -y, -z'
'x-1/2, y, -z-1/2'
'x, -y-1/2, z-1/2'
'-x-1/2, y-1/2, z'
